# Factors influencing COVID-19 vaccine uptake among Latinos: A cross-sectional study

**DOI:** 10.1371/journal.pone.0302332

**Published:** 2024-07-05

**Authors:** Deborah G. Smith, Corey D. Smith, Jennifer A. DeLeon, Jillian L. Sandoz, Carolina O. Ochoa, Martha P. Pearson, Raimunda H. M. Macena

**Affiliations:** 1 Department of Public Health, Louisiana State University Health Science Center Shreveport, Shreveport, Louisiana, United States of America; 2 Department of Medicine, Louisiana State University Health Science Center Shreveport, Shreveport, Louisiana, United States of America; 3 Department of Nursing, Northwestern State University of Louisiana, Shreveport, Louisiana, United States of America; 4 Department of Medicine, Federal University of Ceara, Fortaleza, Ceara, Brazil; Freelance Consultant, Myanmar, MYANMAR

## Abstract

Vaccination against COVID-19 can prevent severe illness and reduce hospitalizations and deaths. Understanding and addressing determinants contributing to vaccine uptake among high-risk groups, such as Latinos, are pivotal in ensuring equitable vaccine distribution, promoting health equity, and fostering community engagement to bridge the gap in vaccine acceptance and ultimately enhance public health. This study aimed to examine factors influencing vaccine uptake among Latinos. We conducted a cross-sectional study using an online platform (n = 242). The survey was administered using a multimodal approach. Strategies for recruitment included community outreach, social media, and targeting community networks serving Latinos. Descriptive statistics, chi-square, and multivariable analysis were performed. Overall, 81.4% of respondents had received at least one dose of the COVID-19 vaccine, with 77.0% recommending it and 70.6% believing it to be safe, 66.7% believing in its efficacy, 62.3% able to find trustful information in Spanish or Portuguese, and almost 40% who relied on health organizations as their primary resource for COVID-19 vaccine information. Factors significantly associated with vaccine uptake included higher education level (*p*<0.001), English level (*p* = 0.023), living in an urban area (*p =* 0.048), having insurance (*p*<0.001), and having a healthcare provider (*p* = 0.007). Furthermore, belief in vaccine safety and efficacy, trust in public health authorities, concerns about COVID-19, the ability to determine true/false vaccine information during the pandemic, and the availability of trustworthy information in Spanish/Portuguese had statistically significant associations (*p*<0.05) with COVID-19 vaccine uptake. COVID-19 vaccine uptake differed based on sociodemographic and other modifiable factors. Our findings emphasize the importance of implementing targeted interventions and culturally sensitive communication strategies to improve vaccination uptake among the Latino community in the United States.

## Introduction

The COVID-19 pandemic has intensified the existing health and social disparities that affect ethnic and racial minority communities and has particularly affected Latino communities in the United States (US). According to the Centers for Disease Control and Prevention (CDC), the Hispanic/Latino population had the highest morbidity and mortality rates among all minority groups in the US [1]. Current data show that 24% of COVID-19 cases are among Latinos, 12.7% among African Americans, and 4.4% among Asians in the United States. Mortality rates are also higher among Latinos compared to other minorities: 16.4% Latinos, 13% African Americans, and 3.2% Asians [2]. Vaccination against COVID-19 can prevent severe illness and reduce hospitalizations and deaths [3]. To achieve equitable and sustainable vaccination rates, it is important to identify determinants that contribute to vaccine uptake among high-risk groups, such as Latinos.

The World Health Organization (WHO) defines vaccine hesitancy as the delay in accepting or refusing vaccines despite the availability of vaccination services [4]. When COVID-19 vaccines became available in December 2020, vaccine uptake among African Americans and Latinos remained low partly due to inequities in vaccine distribution across the US [5]. As of March 25, 2023, the CDC reports that 89.1% of Latinos in the United States have received at least one dose of the COVID-19 vaccine. This vaccination rate is slightly higher than the 87.0% rate among non-Latino White Americans and similar to the 89.2% rate among African Americans [6]. Asian Americans, meanwhile, have the highest vaccination rate at 98.2% [6]. Barriers and facilitators to vaccination may differ across settings and populations depending on the contextual environment, available resources, and prevalent cultural myths, beliefs, and practices [7]. Identifying factors associated with COVID-19 acceptance among minority groups can assist public health authorities in developing effective strategies to improve vaccination uptake.

Developing targeted strategies for COVID-19 vaccine uptake requires understanding social, cultural, and political contexts among different racial/ethnic groups [8]. Ethnic minority groups, including Latinos, often reside in socioeconomically disadvantaged regions, which poses challenges in accessing healthcare [9]. Furthermore, limited education serves as another barrier to healthcare, which is also linked to different vaccination rates across the country among minority groups [10]. Additionally, studies have shown that people from racial and ethnic minority groups have limited access to job opportunities that offer health insurance and paid sick leave [11]. Misinformation, myths, mistrust in the government, political and religious beliefs, and concerns about vaccine safety may also increase public anxiety about vaccines and politicize vaccination policies [12, 13].

The literature has limited information on factors affecting COVID-19 vaccine uptake among Latinos in Northwest Louisiana. Therefore, this study aims to fill this gap by identifying the factors influencing vaccine uptake among the Latino population in Louisiana and providing recommendations to increase vaccination rates. By understanding the underlying barriers and facilitators, we can develop culturally sensitive and effective interventions to achieve health equity for Latino communities. This research holds significant public health implications for mitigating the impact of COVID-19 and ensuring that everyone, regardless of their cultural or ethnic background, has equal access to vaccines and the opportunity to protect their health and well-being. In addition, this study is unique because specifically targets Latinos in Northwest Louisiana. Geographic variations can play a significant role in health-related behaviors and access to healthcare.

## Material and methods

### Study design and setting

A cross-sectional survey was conducted in Northwest Louisiana from June 1, 2022, to March 13, 2023. According to the 2020 United States Census, 26,838 Hispanics or Latinos live in Northwest Louisiana, accounting for 8% of the total population in this area. Northwest Louisiana comprises 13 parishes: Bienville, Bossier, Caddo, Claiborne, DeSoto, Jackson, Lincoln, Natchitoches, Red River, Sabine, Union, Winn, and Webster [14, 15].

### Participants

The target sample size for our study was 200 with a ±5% margin of error and a confidence level of 95%. A total of 242 participants took part in the survey. Eligible criteria included age 18 or older, identifying as Hispanic or Latino in race/ethnicity, and currently residing in Northwest Louisiana.

### Data collection

The survey was administered to participants via the Survey Monkey^®^ platform using non-probabilistic snowball sampling and time-location sampling (TLS) methods. The snowball sampling method relies on existing participants to refer others, helping to include individuals who might not be easily accessible or known to the researchers [16–18]. For instance, we engaged community leaders within the Latino population by inviting them to participate in the survey and encouraging them to extend the invitation to their peers. Additionally, we utilized the snowball sampling approach, leveraging healthcare organizations and local clinics in Northwest Louisiana to promote awareness about the study. These clinics allowed our research team to present information about the study in their waiting room and invite their patients to answer the survey. In addition, we shared the research flyer with patients so they could distribute the survey link and QR code to their family and friends. This method allowed us to obtain a reasonably representative sample of Latinos living in northwest Louisiana. We used different platforms to disseminate the survey, such as Facebook, Instagram, and WhatsApp. The time-location sampling (TLS) method was utilized to obtain the target sample size. The primary aim of TLS is to sample individuals at frequently visited locations, but to avoid undercoverage bias, it is necessary to identify locations that a significant proportion of the target population visits regularly [19]. Before the survey, we identified such locations by contacting nonprofit organizations and community leaders to map specific locations and times where we could reach out to the target population. We conducted community outreach at church events, local health clinics, and Latino markets, distributing flyers and asking eligible people to answer the study survey. Participants were asked to complete a brief anonymous survey available in three languages to ensure inclusivity: Spanish, Portuguese, and English.

### Variables

Data was collected using a structured questionnaire containing the following variables: sociodemographic characteristics, comorbidities, COVID-19-related experiences, COVID-19 testing, perceived risk of infection, COVID-19 vaccination (at least one dose), and the likelihood of accepting COVID-19 vaccine. Participant characteristics in the survey included geographic region, age, sex, country, educational attainment, marital status, religion, and employment. Sex was classified as female or male. Age was classified into three categories (18–24, 25–49, and higher than or equal to 50). We classified the education level into three groups: less than high school, high school or GED, and more than high school. The poverty level was organized into two categories (below and above the poverty line). This variable was calculated based on household size and the U.S. Federal Poverty Guidelines for 2023 [20].

### Data analysis

The collected data were analyzed using the Statistical Package for the Social Sciences^®^ (SPSS), version 28. We used descriptive statistics to summarize and compare the characteristics of the overall sample. We used chi-square tests for categorical variables and t-tests for continuous variables to compare differences in vaccine acceptance by participants’ characteristics and other determinants.

### Ethical approval

This study was approved on March 28, 2022, by the Institutional Review Board (IRB) at Louisiana State University Shreveport (#2021–00029). A written consent was obtained before completing the self-administered questionnaire.

## Results

The survey had 242 participants with an average age of 42.2 years (standard deviation of 12.10 years). Most participants were female, accounting for 64.9% of the group; 56.6% were born in Mexico; 37.7% had completed high school education; and 38.4% reported a lack of English proficiency. The majority, 61.3%, were married; 61.2% lived in an urban area; 51.1% were below the poverty level; and 66.1% didn’t have insurance (Table 1).

**Table 1:** Sociodemographic characteristics of respondents (N = 242).

Variables	N	%
**Parish**		
Bossier Parish	116	47.9%
Caddo Parish	110	45.5%
DeSoto	2	0.8%
Lincoln Parish	1	0.4%
Ouachita Parish	7	2.9%
Rapides Parish	2	0.8%
Union Parish	1	0.4%
Webster Parish	3	1.2%
**Age**		
18–24 yrs	18	7.4%
25–49 yrs	162	66.9%
+50 yrs	62	25.6%
**Sex**		
Female	157	64.9%
Male	85	35.1%
**Country**		
Mexico	137	56.6%
Nicaragua	26	10.7%
United States	20	8.3%
Honduras	16	6.6%
Brazil	17	7.0%
Guatemala	10	4.1%
Argentina	6	2.5%
Colombia	2	0.8%
Peru	2	0.8%
Puerto Rico	2	0.8%
Panama	1	0.4%
El Salvador	2	0.8%
Costa Rica	1	0.4%
**Education** ^a^		
Kindergarten or less	10	4.2%
8th grade or less	35	14.8%
Some high school	46	19.5%
High school/GED	89	37.7%
Bachelor’s degree	9	3.8%
Graduate degree	25	10.6%
Post-graduate degree	22	9.3%
**English level**		
Very well	53	21.9%
Well	58	24%
Not well	93	38.4%
Don’t speak English	38	15.7%
**Marital Status** ^a^		
Married	176	72.7%
Single	36	14.9%
Divorced/Widow	30	12.4%
**Area lives**		
Urban	148	61.2%
Suburb	42	17.4%
Rural	52	21.5%
**Employment** ^a^		
Employed	181	74.8%
Not employed	48	19.8%
Student	10	4.1%
**Religious**		
Yes	165	68.2%
No	77	31.8%
**Annual household income**		
49,999 or less	136	56.2%
50,000 or more	45	18.6%
Not reported	61	25.2%
**Poverty Level**		
Below	95	51.1%
Above	91	48.9%
**Finances in the last 6 months**		
Improved	43	17.8%
Same	153	63.2%
Worse	46	19.0%
**Finances after pandemic**		
Improved	36	14.9%
Same	139	57.4%
Worse	67	27.7%
**Insurance**		
Yes	82	33.9%
No	160	66.1%
**Health Care Provider**		
Yes	101	41.7%
No	141	58.3%

^a^ Due to small cell sizes, participants who selected ‘prefer not to answer’ for education (n = 6), marital status (n = 7), and employment (n = 3) were excluded.

Overall, 81.4% had received the COVID-19 vaccine, with 77.0% recommending it and 70.6% believing it to be safe; 66.7% believed in its efficacy; 62.3% could find trusted information in Spanish/Portuguese; and almost 40% relied on health organizations as their primary resource for COVID-19 vaccine information (Table 2).

**Table 2:** Willingness, beliefs, and awareness towards COVID-19 vaccine among Latinos.

Variables	N	%
**Have you taken the COVID-19 vaccine (at least one dose)?**		
Yes	197	81.4%
No	47	18.6%
**If yes, where would you prefer to receive the vaccine?**		
Clinic/hospital	9	28.1%
Pharmacy	1	3.1%
Work	1	3.1%
Uncertain	21	65.6%
**Would you vaccinate your children?**		
Yes	157	77.0%
No	27	13.2%
Uncertain	20	9.8%
**Do you recommend the vaccine?**		
Yes	157	77.0%
No	25	12.3%
Uncertain	22	10.8%
**Do you believe the vaccine is safe or unsafe?** ^b^		
Safe	144	70.6%
Unsafe	60	29.4%
**Do you believe in the COVID-19 vaccine efficacy?**		
Yes	136	66.7%
No	68	33.3%
**Do you trust Public Health authorities?**		
Not at all	19	9.3%
A little	47	23.0%
Moderately	63	30.9%
Very much	75	36.8%
**Were you able to determine if information about the COVID-19 vaccine was true or false?**		
Yes	135	66.2%
No	69	33.8%
**Can you find trustful information in Spanish or Portuguese?**		
Yes	127	62.3%
No	77	37.7%
**Which social media do you use most?**		
Facebook	135	66.2%
Instagram	17	8.3%
TikTok	6	2.9%
Snap Chat	3	1.5%
Twitter	4	2.0%
Other	39	19.1%
**Information Resources**		
Health Organizations	192	39.8%
Health Professionals	130	27%
Media Sources	85	17.6%
Family or Friends	56	11.6%
Religious leaders	19	3.9%
**Are planning to get the vaccine?** ^c^		
Yes	7	21.2%
No	26	78.8%
**Do you believe the vaccine is safe or unsafe?** ^c^		
Safe	6	18.2%
Unsafe	27	81.8%

^b^ Asked to participants who received at least one dose of the COVID-19 vaccine.

^c^ Asked to participants who did not receive the COVID-19 vaccine.

Factors significantly associated with vaccine uptake included age 50 years or older (*p* = 0.038), a higher education level (*p*<0.001), speaking English well (*p* = 0.027), living in an urban area (*p* = 0.038), having insurance (*p*<0.001), and having a healthcare provider (*p* = 0.009) (Table 3).

**Table 3:** Sociodemographic factors influencing the COVID-19 vaccine uptake among Latinos.

Variables	COVID-19 vaccine				p-value
	Yes		No		
	n	%	n	%	
**Parishes**					0.621
Bossier Parish	97	83.6%	19	16.4%	
Caddo Parish	88	80.0%	22	20.0%	
Other Parishes	12	75.0%	4	25.0%	
**Age (years)**					0.038
18–24	13	72.2%	5	27.8%	
25–49	127	78.4%	35	21.6%	
+ 50	57	91.9%	5	8.1%	
**Sex**					0.072
Female	133	84.7%	24	15.3%	
Male	64	75.3%	21	24.7%	
**Education level**					<0.001
< High School	64	70.3%	27	29.7%	
High School/GED	75	72.4%	14	15.7%	
>High School	53	94.6%	3	5.4%	
**English level**					0.027
Very well	41	77.4%	12	22.6%	
Well	55	94.8%	3	5.2%	
Not well	71	76.3%	22	23.7%	
Don’t speak English	30	78.9%	8	21.1%	
**Marital Status**					0.308
Married	146	83.0%	30	17.0%	
Single	26	72.2%	10	27.8%	
Divorced/Widow	25	83.3%	5	16.7%	
**Area lives**					0.038
Urban	126	85.1%	22	14.9%	
Suburb	35	83.3%	7	16.7%	
Rural	36	69.2%	16	30.8%	
**Employment status**					0.237
Employed	153	80.1%	38	19.9%	
Unemployed	42	87.5%	6	12.5%	
**Religion**					0.341
Yes	137	83.0%	28	17.0%	
No	60	77.9%	17	22.1%	
**Annual household income**					0.107
$49,999 or less	106	77.9%	30	22.1%	
$50,000 or more	40	88.9%	5	11.1%	
**Has insurance**					<0.001
Yes	76	92.7%	6	7.3%	
No	121	75.6%	39	24.4%	
**Has HCP**					0.009
Yes	90	89.1%	6	10.9%	
No	107	75.9%	27	24.1%	

Attitudes and beliefs towards the COVID-19 vaccine also influenced the Latino community in Northwest Louisiana. Several attitudinal factors had statistically significant associations (*p*<0.05) with COVID-19 vaccine uptake: Having been tested for COVID-19, worried about getting COVID-19, believing in vaccine safety and efficacy, trust in public health authorities, concerns about COVID-19, the ability to determine true/false vaccine information during the pandemic, and the availability of trustworthy information in Spanish or Portuguese (Table 4).

**Table 4:** Attitudes and beliefs influencing COVID-19 vaccine uptake among Latinos.

Variables	COVID-19 vaccine				p-value
	Yes		No		
	N	%	n	%	
**Had tested for COVID-19**					0.023
Yes	158	84.5%	29	15.5%	
No	39	70.9%	16	29.1%	
**Had COVID-19**					0.335
Yes	92	77.3%	27	22.7%	
No	85	82.5%	18	17.5%	
**Would you vaccinate your children?**					<0.001
Yes	143	91.1%	14	8.9%	
No	16	59.3%	11	40.7%	
Uncertain	12	60.0%	8	40.0%	
**How worried are you with COVID-19?**					0.032
Unsure	20	100%	0	0%	
Not concerned	53	74.6%	18	25.4%	
Little concerned	76	78.4%	21	21.6%	
Moderately concerned	36	85.7%	6	14.3%	
Very concerned	12	100%	0	0%	
**Do you recommend the COVID-19 vaccine?**					<0.001
Yes	165	92.2%	14	7.8%	
No	15	48.4%	16	51.6%	
**Do you believe the vaccine is safe or unsafe?**					<0.001
Safe	156	96.3%	6	3.7%	
Unsafe	38	51.4%	36	48.6%	
**Do you believe in the COVID-19 vaccine efficacy?**					<0.001
Yes	147	96.1%	6	3.9%	
No	47	56.6%	36	43.4%	
**Do you trust Public Health authorities?**					<0.001
Not at all	7	35.0%	13	65.0%	
A little	39	62.9%	23	37.1%	
Moderately	71	94.7%	4	5.3%	
Very much	77	97.5%	2	2.5%	
**Can you find trustful information in Spanish/Portuguese?**					0.131
Yes	117	85.4%	20	14.6%	
No	77	77.8%	22	22.2%	
**Were you able to determine if information about the COVID-19 vaccine was true or false?**					0.015
Yes	135	86.5%	21	13.5%	
No	59	73.8%	21	26.3%	

## Discussion

Before the COVID-19 vaccine became available, several previous studies conducted in the United States showed that one-third of the adult population reported they would not get it [21, 22]. In these studies, vaccine acceptance was lower for certain racial and ethnic groups, such as Blacks and Latinos [23–25]. Despite the hesitance predicted by those studies, our findings showed that Latinos in Louisiana had a similar vaccination acceptance rate to the national data, where 89.1% of Latinos reported receiving at least one dose of the vaccine [6]. Mass vaccination campaigns and community outreach in Louisiana provided equitable access to COVID-19 vaccines, potentially closing coverage gaps among Latinos [26]. A recent survey by the Kaiser Family Foundation found that 54% of Hispanic adults and 51% of Black adults say they will get the new COVID-19 vaccine, compared to 42% of White adults [27].

Our results also showed that beliefs in vaccine safety and efficacy were associated with increased vaccine uptake among Latinos. Individuals who believed the vaccine to be safe and effective were more likely to get vaccinated, indicating the importance of communication strategies that address vaccine safety concerns. In addition, trust in public health authorities is essential for fostering confidence in vaccination programs [28]. This also coincides with our results, as survey responses that indicated greater trust in public health officials were associated with higher vaccination acceptance. Furthermore, concerns about COVID-19’s morbidity and mortality were associated with increased vaccination uptake [5, 29]. Individuals more concerned about COVID-19 were more likely to get vaccinated, highlighting the role of risk perception in vaccine decision-making [30]. This aligns with our findings that those who were more concerned with COVID-19 had higher vaccination uptake.

This study showed that older adults were more likely to be vaccinated than younger adults, which correlates with other research showing generational differences in vaccination beliefs. This may mean that younger people believe they have better immunity and do not need to be vaccinated or that they are more exposed to vaccine-related misinformation [31]. The 2021 Household Pulse Survey data showed that older adults in the United States had an intent or willingness to become vaccinated once the vaccine was shown to be safe [31]. Studies showed higher vaccine willingness and acceptance among Latinos and older adult groups in the United States, possibly due to risk perception and knowledge about COVID-19 outcomes [5, 32, 33].

Access to healthcare services and information provided by healthcare professionals can positively influence vaccine decision-making [34, 35]. In this study, Latinos with health insurance and a healthcare provider (HCP) were more likely to be vaccinated than those without insurance and HCP. We also found that Latinos living in urban areas were more likely to get vaccinated than those living in suburban or rural areas. This may be partly due to urban areas typically having better access to healthcare facilities and vaccination centers, which may contribute to the higher vaccine uptake in these regions [36]. Additionally, urban areas are more likely to have higher infection rates and a greater awareness of the “risk of mortality,” which can increase fear of disease and a willingness for vaccine uptake, as discussed previously [29].

Educational factors have also been shown to influence COVID-19 vaccination uptake among different racial and ethnic groups in the United States [35, 37]. Our study showed that Latinos with higher education levels were more likely to accept the COVID-19 vaccine than those with lower education attainment. This aligns with previous research, which suggests that individuals with higher education tend to be more health-conscious and possess greater vaccine literacy, enabling them to better understand the benefits of vaccination [38–40].

Cultural factors also influenced vaccination acceptance. Although treated as a homogenous population, every Latino subgroup represents many nations of origin, each being culturally distinct and containing different cultural beliefs about vaccines. Overall, Hispanic and Latinx people are deeply rooted in language, family, and religion. During the COVID-19 pandemic, the value of protecting family members was emphasized through interventions promoting the importance of testing and vaccination [41]. In this population, COVID-19 vaccination coverage was high, including the number of individuals expressing favor regarding vaccination for their children and confidence in recommending the vaccine to others [42]. This reveals a willingness across behavioral characteristics to accept and receive vaccination. Studies showed relatively high acceptance rates among Latinx individuals in Mexico, Brazil, and Ecuador [28, 43]. Hispanic and Latinx people rely on trusted resources for vaccine-related decision-making. These include community members like bilingual health workers and religious groups, as well as authoritative online sources such as the World Health Organization [44, 45]. These trusted sources have served to address information gaps about vaccines, specifically for COVID-19, and strengthen confidence in vaccine safety and importance.

Although progress in vaccine acceptance is evident, continued efforts are still needed to achieve higher vaccination rates for the Latino population in Northwest Louisiana. Even though many Latino people have accepted the COVID-19 vaccine, vaccine hesitancy persists. Prior studies have identified several factors that contribute to this hesitancy, such as mistrust of medical personnel, mistrust of the health system, and medical racism [46]. Requirements of identification and eligibility, mistrust of the government, and anti-immigration policies also play a role in this hesitancy [47]. These are further complicated by inadequate access to language-appropriate and culturally concordant services and information, cost-related concerns, and misconceptions about vaccine side effects [48, 49]. In addition, cultural traditions, such as mistrust of non-natural medicines, lead to a preference for traditional herbal or home remedies. This can contribute to the belief that vaccination is unnecessary.

Studies have shown that there has been an increased willingness and change in attitude towards the COVID-19 vaccine over time [50]. This trend shows promise for public health campaigns to increase vaccine uptake, and implementing evidence-based practices and programs could help increase vaccine coverage [51, 52]. One study in San Francisco demonstrated increased COVID-19 vaccine uptake among Latino populations through a community-centered (neighborhood) vaccination program that included mobilization, vaccination, and activation that addressed and overcame barriers to COVID-19 vaccination [45]. The program utilized social networks to boost vaccination coverage. Most Latino individuals reported vaccine coverage at the site due to its neighborhood location, convenient scheduling, and recommendation by a trusted community member, friend, or family member [45].

Developing targeted strategies for COVID-19 vaccine uptake requires understanding social, cultural, and political contexts among different racial/ethnic groups. Incorporating these in our interventions can help eradicate misconceptions related to vaccination and other health issues. Promoting vaccine uptake among Latino communities in the US requires culturally sensitive and community-specific strategies. These strategies may include: 1. Culturally Tailored Campaigns: Provide information about vaccines in Spanish and Portuguese using culturally relevant messaging and visuals that resonate with Hispanic/Latino cultural values and traditions. In addition, healthcare providers should receive cultural competency training to understand better the needs and concerns of Hispanic/Latino patients. 2. Community Outreach: Organize vaccination and educational events in accessible locations, such as community centers, churches, and schools. Ensure flexible hours to accommodate work schedules. And engage community leaders, organizations, and influencers to disseminate vaccine information. 3: Health Promoters (Promotores de Salud)—Train and empower community health workers (Promotores de Salud) who are trusted community members to provide information, address concerns, and facilitate access to vaccination. 4. Language Services: Provide interpretation services at vaccination sites for those with Limited English Proficiency (LEP). 5. Data Collection: Collect and analyze demographic data to track vaccine uptake within the Hispanic/Latino community. This helps identify areas of improvement and evaluate the effectiveness of strategies. 6. Long-term Engagement: Continue engagement with the community beyond vaccination efforts to address ongoing healthcare needs and build a lasting relationship.

## Limitations

Due to the online nature of our study, we encountered several limitations. First, the survey’s reliance on internet access may have excluded individuals with limited digital literacy, potentially limiting the representation of certain segments within the Latino community in Northwest Louisiana. Additionally, the voluntary nature of the survey could have led to self-selection bias, favoring respondents who were more directly affected by COVID-19 or held stronger opinions, possibly resulting in an overrepresentation of specific subgroups. Moreover, an anonymous Internet survey offered limited control over participant demographics, leading to variations in the study groups’ gender and age distribution, which may not fully represent the entire region’s population. Although logistic regression analysis was not conducted in this study due to the limited sample size, our findings are consistent with previous research on COVID-19 vaccine uptake among Latinos. This strengthens the external validity of our study and provides further support for the importance of addressing vaccine hesitancy and uptake in this population. Another potential limitation of this study was that we did not differentiate between the primary COVID-19 vaccination series and the booster vaccination when collecting responses, both of which were being administered within the study’s timeframe. Factors influencing booster vaccination uptake could have differed from primary vaccination uptake, which may have given additional insight into vaccination uptake challenges. In addition, we did not compare response differences between responses given in 2022 and responses given in 2023, which may have revealed evolving risk factors from one year to the next. Despite these limitations, our study provides valuable insights into the unique perceptions of Latinos towards the COVID-19 vaccine, encouraging future research to explore diverse data collection methods for a more comprehensive understanding.

## Conclusion

Tailoring strategies to the specific needs, beliefs, and challenges of Latino communities is crucial for increasing vaccine uptake and improving public health outcomes. A multi-dimensional approach is needed to improve perceptions around vaccinations in general and COVID-19 shots specifically. Collaboration between healthcare providers, community leaders, and organizations is essential for the success of these efforts.

## Supporting information

S1 Data (ZIP)
